# The Alteration of Irisin—Brain-Derived Neurotrophic Factor Axis Parallels Severity of Distress Disorder in Bronchial Asthma Patients

**DOI:** 10.3389/fnins.2017.00653

**Published:** 2017-11-23

**Authors:** Magdolna E. Szilasi, Krisztian Pak, Laszlo Kardos, Viktoria E. Varga, Ildiko Seres, Angela Mikaczo, Andrea Fodor, Maria Szilasi, Gabor Tajti, Csaba Papp, Rudolf Gesztelyi, Judit Zsuga

**Affiliations:** ^1^Department of Pharmacology and Pharmacotherapy, Faculty of Medicine, University of Debrecen, Debrecen, Hungary; ^2^Institute of Clinical Pharmacology, Infectious Diseases and Allergology, Kenezy Gyula Teaching County Hospital and Outpatient Clinic, University of Debrecen, Debrecen, Hungary; ^3^Department of Internal Medicine, Faculty of Medicine, University of Debrecen, Debrecen, Hungary; ^4^Department of Pulmonology, Faculty of Medicine, University of Debrecen, Debrecen, Hungary; ^5^Department of Health Systems Management and Quality Management for Health Care, Faculty of Public Health, University of Debrecen, Debrecen, Hungary

**Keywords:** irisin, BDNF, distress disorder, bronchial asthma, SGRQ, whole-body plethysmography

## Abstract

Distress disorder (a collective term for generalized anxiety disorder and major depressive disorder) is a well-known co-morbidity of bronchial asthma. The irisin—brain-derived neurotrophic factor (BDNF) axis is a pathway that influences several neurobehavioral mechanisms involved in the pathogenesis of distress disorder. Thus, the aim of the present study was to quantify the serum irisin and BDNF concentrations in order to investigate the possible link between the irisin/BDNF axis and distress disorder in an asthma patient cohort. Data of 167 therapy-controlled asthma patients were analyzed. Demographic, anthropometric, and anamnestic data were collected, routine laboratory parameters supplemented with serum irisin and BDNF levels were determined, pulmonary function test was performed using whole-body plethysmography, and quality of life was quantified by means of the St. George's Respiratory Questionnaire (SGRQ). Correlation analysis as well as simple and multiple linear regression were used to assess the relationship between the irisin level and the Impacts score of SGRQ, which latter is indicative of the presence and severity of distress disorder. We have found a significant, positive linear relationship between the Impacts score and the reciprocal of irisin level. This association was stronger in patients whose BDNF level was higher, and it was weaker (and statistically non-significant) in patients whose BDNF level was lower. Our results indicate that higher serum irisin level together with higher serum BDNF level are associated with milder (or no) distress disorder. This finding suggests that alteration of the irisin/BDNF axis influences the presence and severity of distress disorder in asthma patients.

## Introduction

Generalized anxiety disorder and major depressive disorder, collectively termed distress disorder (Renna et al., 2017), are well-known co-morbidities of bronchial asthma (referred to as asthma hereinafter; Yang et al., 2016). The significance of distress disorder in asthma symptom control, therapeutic adherence and quality of life is well-established and underscored (Global Initiative for Asthma, 2016). Increased prevalence of distress disorder in asthma patients in comparison with that in the general population is also recognized (Goodwin et al., 2003; Global Initiative for Asthma, 2016; Porsbjerg and Menzies-Gow, 2017). For example, analysis of data from the World Mental Health Survey, a cross-national population-based survey including 85,088 participants (of whom 42,697 self-reportedly suffered from asthma), showed that age- and sex-adjusted odds for depressive and anxiety disorders were 1.6 (CI: 1.4, 1.8) and 1.5 (CI: 1.4, 1.7), respectively, when patients with asthma were compared to those without this disease (Scott et al., 2007). Furthermore, a bidirectional relationship seems to exist between asthma and distress disorder, supported by large prospective studies showing robust association between mental disorders in childhood and subsequent evolution of adult onset asthma (Scott et al., 2007; Patten et al., 2008; Loerbroks et al., 2010; Brunner et al., 2014). Conversely, emergence of respiratory symptoms consistent with increased airway hyperreactivity or asthma exacerbations were reported in relation to even transient provocation of depressed or sad mood (Opolski and Wilson, 2005). This two-way relationship suggests the possibility for a cause-and-effect relationship, and warrants the clarification of plausible shared neurobiological pathways underlying this link (Goodwin, 2016).

Recently, narrowed and rigid contextual learning was proposed as a potential common neurobehavioral mechanism that may underlie distress disorder (Renna et al., 2017). Contextual learning is a basic mechanism involved in reinforcement (reward) learning and motivation (Zsuga et al., 2016a,b) to organize cues and their respective contexts (including rewards) into context frames based on their statistical regularities (Bar, 2007). These will serve as the starting point for making forward looking simulations to maximize the sum of future rewards and govern motivated behavior (for an overview see Zsuga et al., 2016b). Characteristic symptoms such as depressive rumination and anxiety have been directly linked to the alteration of contextual learning (Bar, 2009) in distress disorder.

One possible molecular pathway that may link affective constructs (anxiety, rumination) to reinforcement learning is the irisin—brain-derived neurotrophic factor (BDNF) axis, on basis of its potential role in reinforcement learning, given its modulatory effect in structures related to the generation of context frames inherent of reinforcement learning (Zsuga et al., 2016b). Irisin is a contraction-regulated myokine, a highly conservative protein formed by proteolytic cleavage of fibronectin type III domain-containing protein 5 (FNDC5) (Boström et al., 2012). Expression of FNDC5/irisin is most abundant in skeletal muscle and is enhanced by voluntary exercise (Wrann et al., 2013). Upon its release into the systemic circulation, irisin may cause browning of white adipose tissue (Boström et al., 2012) and is able to cross the blood-brain barrier (Phillips et al., 2014). Moreover its expression is substantial in the central nervous system, e.g., midbrain and hippocampus (Phillips et al., 2014), structures fundamentally involved in reinforcement learning (Zsuga et al., 2016a,c). In the CNS irisin can induce the expression of BDNF (Wrann et al., 2013).

BDNF is a neurotrophin that is involved in preserving the plasticity of the brain (Chao et al., 2006) by inducing long-term potentiation and consequent increase of the strength of synaptic connections inherent of reward-related changes and subsequent modified behavior (Yan et al., 2005). BDNF can bidirectionally cross the blood-brain barrier by means of a high-capacity, saturable transport system (Pan et al., 1998). Recently, our group reported the alteration of the irisin/BDNF axis in mood disorders accompanying chronic obstructive pulmonary disease (COPD) (Papp et al., 2017). Others have shown the association of BDNF gene polymorphism with anxiety and depression in asthma and thus suggested that genetic variants may assume a significant role in the pathogenesis of distress disorder in the context of asthma (Yang et al., 2016). Furthermore, the importance of BDNF genotype, by modulating the effect physical exercise has on BDNF secretion was also shown in patients suffering from mild cognitive impairment (Nascimento et al., 2015). This finding further suggests the clinical significance of the potential link between exercise-induced irisin production and consequent BDNF production, pointing to irisin as the humoral mediator linking exercise to BDNF.

Given previous preclinical findings clearly pointing to irisin as the upstream mediator of hippocampal BDNF production (Wrann et al., 2013), elucidation of this potential mechanism in varying clinical settings is warranted. A plausible approach is to investigate a clinical construct that is possibly under the control of the irisin/BDNF axis. Based on findings of others regarding altered contextual learning in distress disorder, a process inherent of reinforcement learning, as well as prior observations pertaining to the potential involvement of BDNF in mood disorders and anxiety (Latsko et al., 2016; Levada et al., 2016; Cagni et al., 2017) assessing the presence and/or severity of distress disorder is feasible. Due to the abundance of clinical observations regarding the complex interplay between asthma and distress disorder we set out to assess the potential interaction between the irisin/BDNF axis and severity of distress disease in our asthma patient cohort.

## Materials and methods

### Study design and protocol

This is a cross-sectional study of asthma patients (*n* = 163) designed in compliance with the STROBE statement for cross-sectional studies (Von Elm et al., 2007). The present study adheres to the principles of the Declaration of Helsinki and was initiated after the approval of the Ethical Committee of the University of Debrecen (DEOEC RKEB/IKEB 3632-2012). Written informed consent was obtained from each participant prior to inclusion.

The recruitment period for the study was from September 1. 2012 to October 15. 2013. Consecutive patients attending the outpatient unit of the Department of Pulmonology (University of Debrecen) diagnosed with chronic inflammatory airway diseases (asthma, COPD, ACOS, and allergic rhinitis) were screened by pulmonologists blinded to the research hypothesis. Exclusion criteria included history of malignant or benign tumors, or presence of acute inflammation of any kind during the preceding month. During the recruitment period 319 patients were included (asthma: *n* = 167, COPD: *n* = 74, ACOS: *n* = 21, allergic rhinitis: *n* = 56), using the same protocol.

The current study assesses the potential involvement of altered irisin/BDNF axis in distress disorder among in our asthma cohort (*n* = 167). All patients participated in a control-based asthma management program complying with GINA as well as the relevant Hungarian practice guideline (Boulet et al., 2012; Tamási et al., 2012). Treatment at the time of inclusion was provided as clinically warranted hence most patients were regularly using inhaled corticosteroid preparations (*n* = 156) and/or long-acting β_2_ agonists (*n* = 141). Corticosteroid therapy was omitted only due to co-morbidities rendering the risks related to this therapy higher than the accrued benefits. Demographic, anthropometric, and anamnestic (including history of smoking, diabetes, dyslipidemia, and hypertension) data were obtained. Smoking exposure was characterized by pack-years (that included past smoking and current smoking exposure). Disease-specific quality of life was characterized by using the official Hungarian version of the St. George's Respiratory Questionnaire (SGRQ) (Meguro et al., 2007) after obtaining written permission from its proprietor (Paul Jones, University of London, London, UK, on August 28. 2012).

### Pulmonary function testing

Lung function was assessed using whole-body plethysmography, conducted in compliance with the ATS/ERS criteria (Stocks et al., 2001; Miller et al., 2005; Wanger et al., 2005) (Piston whole-body plethysmograph PDT-111/p, Piston Medical, Hungary, equipped with automatic BTPS correction for cabin temperature, humidity and pressure as well as full automatic calibration and leakage test). Patients were asked to take their medication as usual on the morning of their examination (thus plethysmography was done with patients being on asthma control therapy). Best of three technically sound maneuvers was analyzed for each participant. At least two separate and technically appropriate measurements were conducted for resistance curves (each measurement consists recordings of at least 5 resistance loops) and results were accepted only if they were the same for both measurements. Raw pulmonary function data and percent predicted of normal reference values were recorded using algorithms provided by the manufacturer. The following parameters were included in the statistical analysis: R_aw_, FEV1% pred, FVC% pred, FEV1/FVC% pred, FEF25-75% % pred, RV% pred, TLC% pred, RV/TLC% pred.

### Blood samples

Routine laboratory investigations were performed on the morning of the study after an overnight fast, according to the standard operational procedures of the Department of Laboratory Medicine (University of Debrecen) as described previously (Papp et al., 2017). Briefly, serum or plasma samples were used to determine parameters descriptive of carbohydrate and lipid homeostasis, of kidney, liver, and muscle function, and of inflammation. CRP was dichotomized as high vs. normal using the cutoff of 4.6 and 5.2 mg/L for female and male patients, respectively. HOMA index was computed as previously (Zsuga et al., 2007). Serum samples for the determination of irisin and BDNF were frozen within 60 min and stored at −80°C until further analysis.

### Determination of serum irisin and BDNF

Serum irisin levels were determined with a commercially available ELISA kit (Phoenix Pharmaceuticals, Burlingame, CA, USA), compliant of the manufacturer's instructions. Summarizing, 50 μl of standard or sample (diluted 2 times), 25 μl primary antibody, and 25 μl biotinylated peptide were added to each well and were incubated at room temperature for 2 h. Next, the plates were washed four times and 100 μl/well SA-HRP solution was added for an hour-long incubation at room temperature. After washing, 100 μl/well of substrate solution was added and incubated for another 1 h, following which 100 μl/well of 2 N HCl was used to terminate the reaction. Absorbance was read immediately at 450 nm. The irisin standard curve was linear from 1.34 to 29.0 ng/ml, and the detection limit was 1.34 ng/ml, in accordance with the information provided by the manufacturer.

Similarly, serum BDNF levels were measured in agreement with the manufacturer's instructions (Sigma-Aldrich, MO, USA). Briefly, standards and samples (diluted 100-fold) were administered into 96-well microplates coated with anti-BDNF monoclonal antibody and incubated at 4°C overnight. After this step, the plates were washed 4 times, and 100 μl biotinylated anti-human BDNF Detector Antibody was added to each well. Plates were incubated with gentle shaking for 1 h. Next, the wells were washed and 100 μl HRP-Streptavidin solution was added to each well that was followed by a 45-min long incubation period at room temperature with gentle shaking. Samples were washed again, then to induce a color reaction 100 μl TMB One-Step Substrate Reagent was added to each well and incubation continued for 30 min. The reaction was stopped using the stop solution supplied by the manufacturer. The absorbance at 450 nm was measured immediately with an automated microplate reader. The detection limit for BDNF was <80 pg/ml.

A standard curve showing linear relationship between optical density and concentration of irisin as well as BDNF were obtained with each plate. All measurements were performed in duplicate.

### St. George's respiratory questionnaire (SGRQ)

The Hungarian version of SGRQ validated for a 1-month recall period was delivered using supervised self-administration, according to the SGRQ manual supplied by the copyright holder (Jones et al., 1991) and as described previously (Papp et al., 2017). SGRQ yields a Total score descriptive of the overall influence of the disease in terms of health status and three component scores, the Symptoms, the Activity, and the Impacts score. The main outcome measure of interest was the SGRQ Impacts score, as it characterizes disturbances of the psycho-social functioning, and thereby shows strong correlation with distress disorder (Jones et al., 1991). Scores are expressed as a percentage, with 100% representing the worst and 0% indicating the best possible subjective health status. Differences in scores were considered clinically meaningful if they exceeded 4 percent points (Jones, 2005). Data entry was performed by two independent raters using a proprietor-supplied SGRQ calculator (Ferrer et al., 2002). Raters were blinded to patients' irisin and BDNF levels. Inter-rater variability assessed by Spearman correlation provided coefficients of 0.976 (*p* < 0.001), 0.997 (*p* < 0.001), 0.998 (*p* < 0.001), and 0.998 (*p* < 0.001) for the Symptoms, Activity, Impacts and Total scores, respectively (Impacts score was unavailable for one patient). The mean of scores obtained by the independent raters was used for statistical analysis.

### Statistical analysis

Normality for continuous variables was checked with Shapiro–Wilk test. If distribution was Gaussian, Student's *t-*test was used for the comparison of two data sets (and data were presented as mean ± *SD*), if not, Mann–Whitney *U-*test was performed (and data were presented as median; interquartile range). Frequencies were compared with Pearson's χ^2^ test.

Descriptive statistics were provided for the whole data set and the data dichotomized according to the median of two parameters, the Impacts score (as cutoff value). Patients with Impacts score <22.53% and ≥22.53% were categorized into the lower (*n* = 84) and higher Impacts score group (*n* = 83), corresponding to the presence of less (or no) and more severe distress disorder, respectively.

The correlation between the Impacts score and (transformed) serum irisin level was established with Pearson's correlation in the whole data set as well as in the lower and higher BDNF groups. Serum irisin values underwent a reciprocal transformation to ensure Gaussian distribution.

In order to account for potential confounders, multiple regression modeling was carried out. This procedure was initiated by assessing normality of each variable. Values of HgA1c, HOMA index, CK, HDL-cholesterol, triglyceride, sTSH, and R_aw_ were log-transformed, furthermore reciprocal of urea and reciprocal of square of glucose concentration were computed to get hold of the Gaussian distribution of variables. Simple linear regression analysis was performed to identify possible determinants of the Impacts score (characteristic of distress disorder) as well as the (reciprocal of) serum irisin. The influence of traditional confounding factors (age, gender, height) as well as disease duration were assessed. Missing data were omitted. Simple linear regression revealed interaction between height and weight squared. After the simple regression, age, gender, height (in interaction with weight squared) (as *a priori* variables) as well as all significant regressors were introduced into a multiple linear regression model to further quantify the relationship between the Impacts score and serum irisin concentration. Other SGRQ scores were omitted due to their high collinearity with the Impacts score. Lung function parameters yielding significant regressors were combined into a single parameter descriptive of lung function using principal component analysis. Variables were entered in the model simultaneously, and then factors not significantly contributing to the model were deleted. (Eventually, the final model contained all variables identified *a priori*, the composite of lung function parameters and the history of dyslipidemia.) In addition, the final model was stratified with respect to the median of BDNF. Patients with BDNF level <311.4 μmol/L and ≥311.4 μmol/L were categorized into the lower (*n* = 84) and higher BDNF group (*n* = 83), respectively. Heteroskedasticity and goodness of fit for the model was assessed by Cook-Weisberg and Ramsey tests, respectively.

Statistical analysis was performed with Stata 13.0 software (Stata Corporation). Values are given as mean ± *SD* or medians (with interquartile ranges: IQR), excepting regression coefficients which are presented with their 95% confidence intervals (CI).

## Results

### Patients

The basic characteristics of our asthma patient cohort (*n* = 167) were previously described (Tajti et al., accepted). The median irisin level (IQR) was 7.87 (7.15–8.82) ng/mL in the whole sample, and it was 8.03 (7.14–9.50) ng/mL, and 7.86 (7.15–8.73) ng/mL, among male and female patients, respectively. The mean of serum ± BDNF level was 314.46 ± 118.68 ng/mL in the whole sample and it was 300.07 ± 107.58 and 326.48 ± 126.55 ng/mL among men and women, respectively. No statistical difference was present in either serum irisin or serum BDNF levels with respect to gender. The main demographic, anthropometric and anamnestic data, pulmonary function, laboratory and SGRQ parameters are summarized in Table 1.

**Table 1:** The main characteristics of the asthma cohort (*n* = 167).

**Parameters**	
Age (years)	48 (36–58)
Gender (f/m)	91/76
Smoker (n/y)	145/22
Smoking (years)	0 (0–1)
Smoking (pack-years)	0 (0–3.375)
Diabetes (n/y)	159/8
Dyslipidemia (n/y)	118/49
RR systolic (mmHg)	131.56 ± 15.24
RR diastolic (mmHg)	84.31 ± 10.68
Hypertension (n/y)	104/63
Disease duration (years)	15 (10–20)
Waist (cm)	96.37 ± 13.07
Weight (kg)	75 (65–88)
Height (m)	1.68 ± 0.10
Irisin (ng/mL)	7.87 (7.15–8.82)
BDNF (ng/mL)	314.46 ± 118.68
Urea (mmol/L)	4.4 (3.9–5.5)
Creatinine (μmol/L)	69 (58–79)
GFR (ml/min/1.73 m^2^)	91 (85–91)
GOT (U/L)	19.5 (16–25)
GPT (U/L)	19 (14–29)
γGT (U/L)	22 (16–33)
CK (U/L)	111.5 (81–160)
LDH (U/L)	196 (180–224)
Glucose (mmol/L)	5 (4.3–5.4)
Insulin (mU/L)	9.1 (6.3–17)
HgA1C (%)	5.4 (5–5.8)
HOMA	2.00 (1.30–4.13)
Cholesterol (mmol/L)	5.30 ± 1.18
LDL-C (mmol/L)	3.16 ± 0.93
HDL-C (mmol/L)	1.4 (1.2–1.8)
Apo-A1 (g/L)	1.59 ± 0.29
ApoB (g/L)	0.99 (0.85–1.18)
Lp(a) (mg/L)	122.5 (52–376)
TG (mmol/L)	1.3 (0.9–2)
CRP (high/low)	30/136
Fibrinogen (g/L)	3.33 ± 0.64
Procalcitonin (μg/L)	0 (0–0)
Steroid use (n/y)	11/156
SGRQ symptoms score	28.65 (11.70–51.75)
SGRQ impacts score	22.53 (8.93–38.44)
SGRQ activity score	47.24 (23.61–59.45)
SGRQ total score	32.16 (17.10–47.11)

*Data are presented as mean ± SD or median (interquartile range) unless otherwise stated. f/m, female/male; y/n, yes/no; SGRQ, St. George's Respiratory Questionnaire*.

### Comparison of patients regarding distress disorder

The two strata of our asthma cohort, dichotomized based on the median of Impacts score (reflective of the severity of distress disorder present), were homogenous regarding most parameters investigated (Table 2). Nonetheless, patients with more severe form of distress disorder (i.e., those in the higher Impacts score group) were significantly older, shorter, had smoked more (reflected by pack-years), and had higher serum triglyceride and fibrinogen but lower irisin levels. In addition, they performed poorer in the lung function test reflected by worse parameters in terms of FVC% pred, FEV1% pred, FEF25-75% pred %, RV% pred, RV/TLC% pred, and R_aw_, and had significantly higher Symptoms, Activity and (as a consequence) Total scores. The difference of scores was clinically meaningful, e.g., it was higher than 4 points (Jones, 2005). Furthermore, higher proportion of patients in the higher Impacts score group suffered from hypertension and dyslipidemia (Table 2).

**Table 2:** Main characteristics of two groups of the asthma cohort dichotomized according to the severity of distress disorder indicated by the SGRQ's Impacts score.

**Parameters**	**Lower impacts score**	**Higher impacts score**	***p***
Age (years)	40.5 (28.5–59.5)	52 (43–57)	0.003
Smoking (pack-years)	0 (0–0.5)	0 (0–5)	0.03
Dyslipidemia present (n/y)	67/17	51/32	0.009
Hypertension present (n/y)	62/22	42/41	0.002
Height (m)	1.70 ± 0.10	1.66 ± 0.10	0.003
Irisin (ng/mL)	8.187 (7.402–9.312)	7.666 (6.838–8.54)	0.02
Triglyceride (mmol/L)	1.2 (0.9–1.8)	1.6 (1–2)	0.04
Fibrinogen (g/L)	3.18 ± 0.62	3.49 ± 0.62	0.001
FVC % pred	95.33 ± 13.59	90.05 ± 13.58	0.02
FEV1 % pred	90.27 ± 13.86	82.33 ± 15.20	<0.001
FEF25-75 % pred	72.56 ± 22.06	61.11 ± 21.05	0.001
RV % pred	129 (112–146)	139 (124−169)	0.01
RV/TLC % pred	117.60 ± 19.11	129.46 ± 19.32	<0.001
R_aw_ current	0.2 (0.17–0.25)	0.24 (0.19–0.32)	0.003
**Symptom score**	**15.43 (8.40–26.87)**	**50.46 (31.44–66.22)**	**<0.001**
**Activity score**	**29.38 (11.56–42.82)**	**59.45 (47.68–66.95)**	**<0.001**
**Total score**	**17.10 (9.22–25.57)**	**47.11 (37.88–56.10)**	**<0.001**

*The lower (n = 84) and higher (n = 83) Impacts score group had Impacts score <22.53 or ≥22.53, respectively. Only parameters with significant difference are indicated. Comparison of smoking (in years), presence or absence of diabetes, myocardial infarct, stroke, and steroid use in case history, RR systolic (mmHg), RR diastolic (mmHg), disease duration (years), waist (cm), weight (kg), BDNF (ng/mL), urea (mmol/L), creatinine (μmol/L), GFR (mL/min/1.73 m2), GOT (U/L), GPT (U/L), γGT (U/L), CK (U/L), LDH (U/L), glucose (mmol/L), insulin (mU/L), HgA1c (%), HOMA index, cholesterol (mmol/L), LDL-C (mmol/L), HDL-C (mmol/L), CRP (mg/L), CRP kat (low/high), procalcitonin (μg/L), sTSH (mU/L) showed no significant difference between the two groups. Data are presented as mean ± SD or median (interquartile range), unless otherwise stated. Differences between the two groups were considered significant at p < 0.05, clinically significant difference between component scores are indicated in bold*.

### Associations between SGRQ's impacts score, serum irisin, and BDNF levels

**Figure 1:**
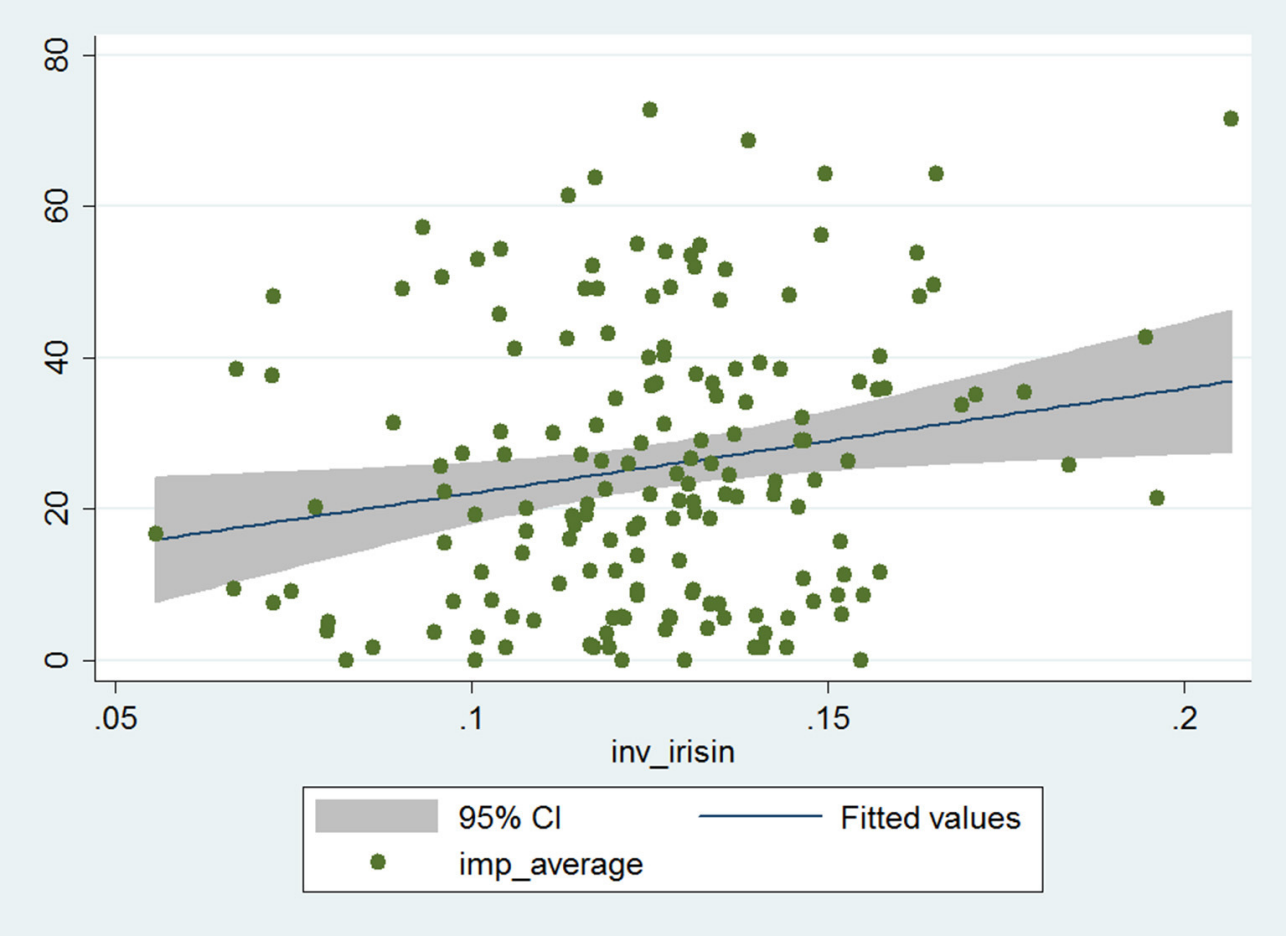
Correlation of the Impacts score of SGRQ and reciprocal of serum irisin concentration in the whole data set (*n* = 167). The gray zone indicates the 95% confidence interval, while the blue line (in it) shows the fitted values of the reciprocal of irisin and Impacts score data pairs.

The Impacts score and reciprocal of serum irisin level showed significant positive correlation in the whole patient cohort (Pearson correlation coefficient: 0.19, *p* = 0.014; Figure 1). The correlation coefficient increased indicating stronger correlation (and, despite the smaller patient number, remained statistically significant) in the stratum with higher BDNF levels, while it decreased (and lost its statistical significance) in the lower BDNF group (Pearson correlation coefficient: 0.25 and 0.11, *p* = 0.025 and *p* = 0.30, respectively). This suggests that serum irisin level is associated with processes that lead to disease-related impairment of psycho-social function among asthma patients, an effect that is more pronounced if higher serum irisin concentration is accompanied with higher serum BDNF level.

**Figure 2:**
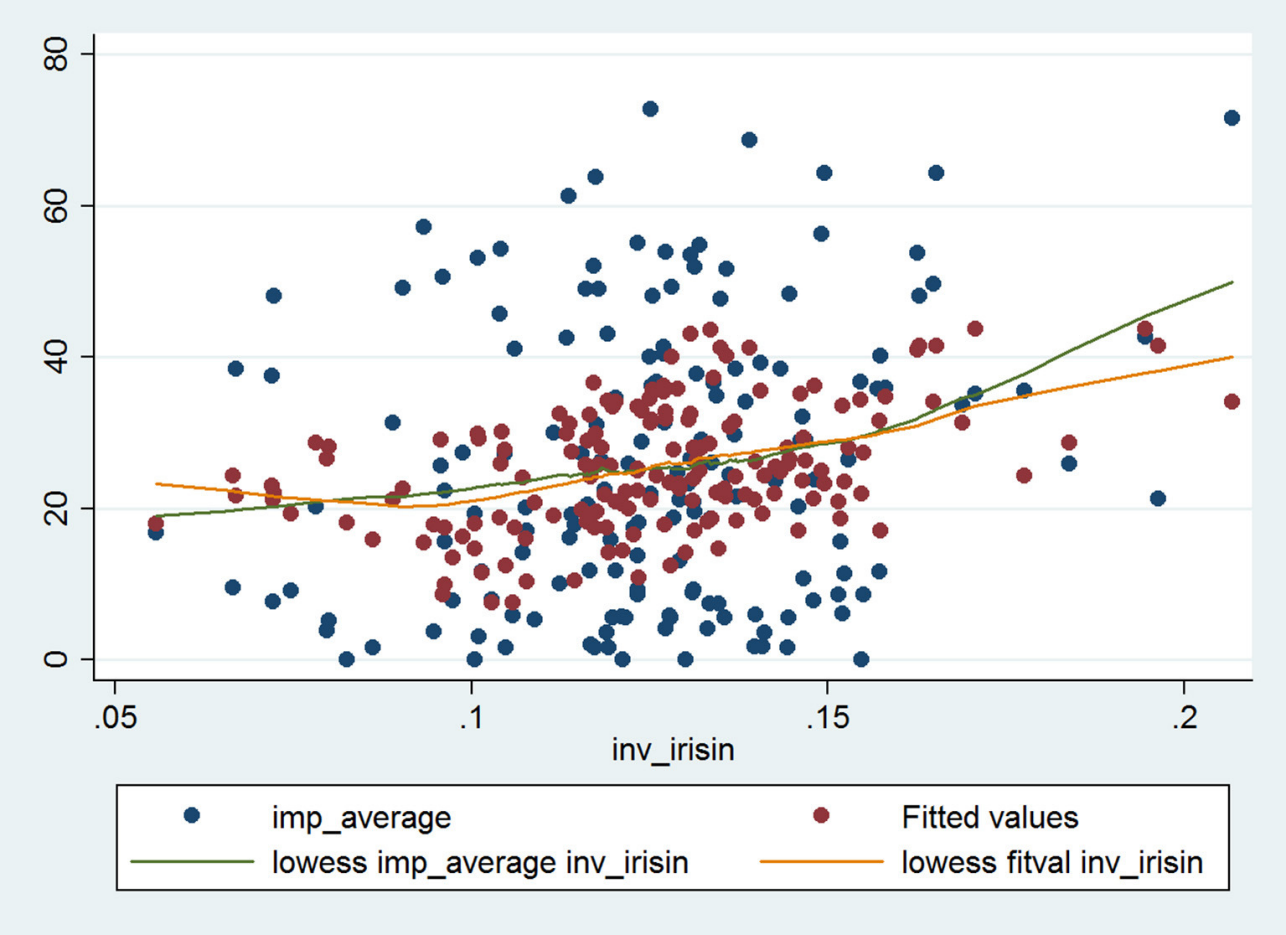
The final multiple linear regression model characterizing the relationship between Impacts score of SGRQ and reciprocal of serum irisin concentration in the whole data set (*n* = 165; data of two patients were not complete). The blue dots indicate the raw (i.e., original) data, while the red dots indicate the fitted values obtained by multiple linear regression. The green and orange lines indicate the fitted curves for raw data and for data provided by multiple regression. The fitted curves were obtained by locally weighted scatterplot smoothing (lowess).

Simple linear regression yielded corroborating results upon evaluating the relationship between Impacts score and reciprocal of irisin (Table 3). This relationship remained significant after adjusting for all significant predictors and *a priori* determinants by means of multiple linear regression. The Impacts score and reciprocal of irisin showed a strong, significant, positive association (β: 147.74; CI: 42.17, 253.30; *p* = 0.006), which was even more pronounced (e.g., the regression coefficient was higher) in the higher BDNF group (β: 213.38; CI: 56.38, 370.38; *p* = 0.008), while it was weaker (an effect reflected by lower regression coefficient and lack of statistical significance) in the lower BDNF group (β: 60.89; CI: −108.55, 230.33; *p* = 0.48). All models (the full model as well as the stratified ones) were significant (*p* < 0.001, *p* = 0.040, *p* = 0.003). The Cook-Weisberg test showed no heteroskedasticity for the full model and strata with higher and lower BDNF levels (*p* = 0.55, *p* = 0.22, and *p* = 0.79, respectively). Moreover, all three models showed good fit reflected by the locally weighted scatterplot smoothing (Figure 2) as well as by the Ramsey test (*p* = 0.51, *p* = 0.28, and *p* = 0.55 for the whole data set and strata with higher and lower BDNF concentrations, respectively).

**Table 3:** Significant predictors of the SGRQ's Impacts score and the reciprocal of serum irisin levels determined using simple linear regression for the whole data set (*n* = 165; data of two patients were not complete).

**Parameter**	**Coefficient (95% CI)**	***p***
**SIMPLE LINEAR REGRESSION OF IMPACTS SCORE**		
Age	0.30 (0.122, 0.49)	0.001
Inverse of irisin	138.54 (28.47, 248.61)	0.014
Height	−50.58 (−78.01, −23.15)	<0.01
Albumin	−1.43 (−2.40, −0.47)	0.004
Log CK	−6.27 (−11.72, −0.83)	0.024
Log TG	5.10 (0.08, 10.12)	0.046
Fibrinogen	5.41 (1.07, 9.75)	0.015
FVC % pred	−0.31 (−0.52, −0.11)	0.003
FEV1% pred	−0.33 (−0.52, −0.15)	0.001
FEF25-75% % pred	−0.18 (−0.31, −0.05)	0.005
RV % pred	0.09 (0.0002, 0.17)	0.049
RV/TLC % pred	0.28 (0.14, 0.42)	<0.001
Log R_aw_	8.98 (1.91, 16.05)	0.013
Symptoms score	0.52 (0.44, 0.61)	<0.001
Activity score	0.56 (0.47, 0.65)	<0.001
Total score	0.94 (0.90, 0.99)	<0.001
Dyslipidemia present	9.72 (3.75, 15.70)	0.002
Hypertension present	8.36 (2.71, 14.00)	0.004
Atherosclerosis present	13.32 (2.20, 24.44)	0.019
Obesity present	8.20 (2.47, 13.93)	0.005
Steroid use	11.23 (0.06, 22.41)	0.049
GFR	6.39 (0.55, 12.24)	0.032
**SIMPLE LINEAR REGRESSION OF RECIPROCAL OF IRISIN**		
Lp(a)	1.39·10^−5^ (1.89·10^−6^, 2.59·10^−5^)	0.024
Impacts score	2.6·10^−4^ (5.35·10^−5^, 4.67·10^−4^)	0.014
Total score	2.17·10^−4^ (1.04·10^−5^, 4.24·10^−4^)	0.040
Myocardial infarct present	0.03 (0.009, 0.057)	0.008
Steroid use	0.02 (0.002, 0.032)	0.029

*Regression coefficient values are presented with their 95% confidence limits*.

The final multiple linear regression model (built for the Impacts score) contained only dyslipidemia in addition to the reciprocal of irisin and *a priori* identified parameters. The presence of dyslipidemia showed a significant, positive association with the Impacts score of asthma patients (β: 7.01; CI: 0.85, 13.35; *p* = 0.026), a relationship that was only significant in the whole data set.

## Discussion

Our major finding is that severity of distress disorder (characterized by the Impacts score of SGRQ) showed robust positive linear relationship with the reciprocal of serum irisin among adult asthma patients, undertaking asthma controller therapy (including, for most patients, an inhaled corticosteroid). Interestingly, severity of distress disorder was more closely associated with the (reciprocal of) serum irisin concentration in the patient stratum with higher BDNF levels, while this association became statistically non-significant in the stratum with lower BDNF levels. The linear relationship remained significant following adjustment for *a priori* variables and potential confounders by means of multiple linear regression. In addition, the final model identified the presence of dyslipidemia as a significant regressor influencing the Impacts score. Quantification of serum irisin levels in asthma for the first time may also be considered as a merit of the study.

Our main finding, that the positive linear relationship between the Impacts score reflective of distress disorder and the (reciprocal of) serum irisin varies with respect to the BDNF level, implicates the possibility of an interaction between these two factors in the context of distress disorder. This finding corroborates our previous hypothesis that serum irisin, via BDNF, may influence reinforcement learning and allied processes including contextual learning (Zsuga et al., 2016c). Furthermore, it is in agreement with our previous report, which have also shown the alteration of irisin/BDNF axis in connection with mood disturbances among COPD patients (Papp et al., 2017). Based on the original report of Wrann et al. (2013) reporting that both FNDC5/irisin is able to induce the expression of BDNF in the hippocampus, several reviews discuss the probable involvement of FNDC5/irisin/BDNF axis in brain health (Chen et al., 2016; Ryan and Nolan, 2016; Camandola and Mattson, 2017; Satoh et al., 2017). Nonetheless, clinical reports also show some inconclusive findings with respect to the relationship between BDNF and irisin levels. For example, in a small clinical sample comprising of endurance athletes and sedentary controls, irisin and BDNF levels as well as metrics for cognitive performance were higher in the athletes' group (Belviranli et al., 2016). Yet in a different study, 3-month long CrossFit training was shown to increase aerobic capacity and BDNF levels but not irisin levels in a group of young physically active volunteers (Murawska-Cialowicz et al., 2015). Nevertheless, the controversies regarding the role irisin may assume in neuronal health and need for further clinical research were also articulated previously (Farshbaf et al., 2016).

Generalized anxiety disorder and major depressive disorder are considered as distinct entities according to nosological systems, classification schemes which have been criticized for being agnostic for underlying etiological factors (Renna et al., 2017). Nevertheless, acknowledging the high co-morbidity for anxiety and depressive disorders, the new Diagnostic and Statistical Manual of Mental Disorders (DSM-V) defines a combined anxiety and depression category (Wegner et al., 2014). Convergence of previously distinct entities in such phenomenological nosological systems is well aligned with the need of identifying common neurobiological pathways involved in the etiopathogenesis of distress disorder (Renna et al., 2017). Linking these psychiatric disorders to the theoretical framework of the proactive model of reinforcement learning acknowledges the possibility that these disorders may evolve due to the disruption of the same core system.

Reinforcement learning is a central process that guides individual behavior in a way that immediate and cumulated future rewards are maximized in the long term (Maia, 2009). The brain has two fundamentally different, but closely interconnected systems governing model-based and model-free reinforcement learning, paradigms that differ in terms of use of a world model. Model-based reinforcement utilizes contextual learning to build a world model by the function of the hippocampus, amygdala and medial orbitofrontal cortex (mOFC) (Zsuga et al., 2016a,b). On the other hand, model-free reinforcement learning samples the environment to obtain current information and compares it to cached values to emit the canonical reward prediction error signal, the function of dopaminerg neurons emanating from the ventral tegmental area (VTA) (Schultz et al., 1997; Schultz, 2007). The mutual influence of these systems has been implicated previously as the dopaminergic reward prediction error signal may contribute to updating the reward attribute of the context frames (by means of VTA's dopaminergic input on the hippocampus, amygdala and mOFC), furthermore glutaminergic outflow of mOFC modulates the function of model-free structures including VTA (Zsuga et al., 2016a,b). Importantly, alteration of several attributes of reinforcement learning (e.g., learning rate, primary reward sensitivity) has been described in relation with both anxiety and depression (Huys et al., 2013; Treadway et al., 2013; Pulcu et al., 2014).

Irisin and its downstream mediator BDNF are humoral factors that target several structures (e.g., hippocampus, amygdala, VTA) and, accordingly, may modulate reinforcement learning (Zsuga et al., 2016c). Irisin, either of peripheral or neuronal origin (Wrann et al., 2013), has the ability to trigger the expression of BDNF in these structures (Wrann et al., 2013; Camandola and Mattson, 2017). The exact molecular mechanism is yet to be identified (Xu, 2013; Camandola and Mattson, 2017). BDNF regulates dopamine 3 (D3) signaling pathways in VTA (Guillin et al., 2003; Collo et al., 2014), a pathway that has fundamental influence on dopamine's effect with respect to structural plasticity in the model-free reinforcement learning system (Schultz, 2007). Furthermore, high expression of BDNF parallels enhancement of long-term potentiation underlying synaptic plasticity, neurogenesis, and neuronal differentiation in the model-based system's amygdala, hippocampus, and prefrontal cortex (Chen et al., 2016; Camandola and Mattson, 2017). Summarizing, the irisin/BDNF axis modulates several structures constituting the neurobiological system underlying reinforcement learning.

The role of BDNF in distress disorder is established to the extent that BDNF is emerging as a biomarker (Polyakova et al., 2015; Ihara et al., 2016). Decreased BDNF levels were shown to parallel progression of depression, inhibit neurogenesis, while causing structural and functional changes in the hippocampus (Jeon and Kim, 2016). Conversely, antidepressant therapy was shown to increase BDNF levels and promoted the recovery of hippocampal nerve cells and improved plasticity (Groves, 2007; Jeon and Kim, 2016). Accumulating evidence led to the formalization of the neurotrophic theory of depression (Duman and Monteggia, 2006; Bus et al., 2015). For example, a systematic review and meta-analysis of 20 studies including 1,054 patients provided robust support for the theory as the authors concluded that BDNF concentration correlated with depression score changes, and improvement of depression due to antidepressant therapy is associated with neuroplastic changes and increased BDNF levels (Brunoni et al., 2008). The presence of longitudinal association between serum BDNF and depression further supports BDNF's significance. Bus et al reported that baseline BDNF levels showed a substantial decline in previously depressed and remitted patients at a 2 years' follow-up (Bus et al., 2015). Complexity of BDNF's influence is getting more and more acknowledged since the contrasting consequences of changes in neuronal plasticity emerge dependent on the involvement of distinct pathways (e.g., hippocampal or VTA/ventral striatal ones; Groves, 2007). Preclinical models showed that increasing influence of BDNF in VTA and ventral striatum may induce depression (Jeon and Kim, 2016). Thus, it was previously suggested that BDNF may act as a critical modulator of the activity-dependent plasticity of distinct neural networks (Allen and Dawbarn, 2006; Farshbaf et al., 2016).

In our previous work, we investigated the possible involvement of the irisin/BDNF axis in the evolution of distress disorder (also quantified by the Impacts component score of SGRQ) in a COPD patient cohort (Papp et al., 2017). Although, similarly to our current findings in asthma, a significant linear relationship was present between the Impacts score and reciprocal of serum irisin levels among COPD patients (β: 419.97; CI: 204.31, 635.63; *p* < 0.001), the interaction between serum irisin and BDNF levels was found to be different. In the case of COPD patients, the linear association became stronger in the stratum of patients with BDNF levels in the lower half (β: 434.11; 95% CI: 166.17, 702.05; *p* = 0.002; Papp et al., 2017). On contrary, results in our cohort of asthma patients in the present study showed more robust linear association in the stratum comprising patients in the upper half of the BDNF levels.

Several causes may underlie this difference. On one hand, the different role of BDNF in the etiopathogenesis of these two similar, but distinct disease entities must be acknowledged. The ability of BDNF to induce airflow limitation and to promote airway hyperresponsiveness to histamine has been described in animal models of asthma as well as in asthma patients (Lommatzsch et al., 2005a, 2009; Prakash and Martin, 2014). Increased neuronal activity and sensitivity underlying these symptoms have been linked to upregulation of BDNF (Lommatzsch et al., 2003; Braun et al., 2004). Meanwhile in COPD, BDNF's role in airway pathology was only implicated in the context of airway remodeling (Pera et al., 2014). On the other hand, the fact that both patient populations were treated as clinically warranted may also carry distinct implications. In the management of asthma inhaled corticosteroid therapy is the preferred controller choice from step 1 and β_2_ agonists are considered only as reliever therapy in steps 1 and 2 and are introduced as controllers from step 3 (Global Initiative for Asthma, 2016). On the other hand, β_2_ agonists are the first choice of therapy from the early stages of stable COPD (e.g., GOLD 1 and GOLD 2 stage) with corticosteroid use being indicated from the more advanced (GOLD 3 and GOLD 4) severity stages (Vogelmeier et al., 2017). Prior clinical reports have shown the ability of β_2_ agonists to increase BDNF concentration in platelets and (consequently) serum, parallel to the deterioration of airway responsiveness (Lommatzsch et al., 2009). Moreover, the causative effect of BDNF in the paradoxical loss of asthma control following unbalanced use of β_2_ agonists has been proposed (Hogg et al., 2004; Nelson et al., 2006). This deleterious effect may be counterbalanced by co-administration of inhaled corticosteroids (Lommatzsch et al., 2009). Based on these considerations, differential alteration of BDNF-related mechanisms in these two diseases seems plausible, although further investigations are needed.

Limited number of reports are available concerning the change of serum BDNF levels in asthma. While the serum BDNF levels reported in the current investigation exceed that of Virchow's group investigating asthma patients (Lommatzsch et al., 2005a,b), they are within the range measured in other studies involving healthy individuals and other patient populations, with reported serum BDNF levels spanning over four orders of magnitude (!), ranging from 0.005 to 280 ng/ml using different ELISA kits (Failla et al., 2016; Ihara et al., 2016; Jacoby et al., 2016; Kheirouri et al., 2016). At the same time, to the best of our knowledge, no previous report is available regarding the serum irisin concentrations in asthma patients. Nevertheless, investigations dealing with other populations showed comparable irisin levels: 26.3 (IQR: 22.6, 32.4) ng/ml, 53.7 (IQR: 46.7, 62.8) ng/ml, 58.5 (42.8, 78.9) ng/ml in smokers with and without COPD, and in non-smoking individuals, respectively (Kureya et al., 2016).

In the current study, based on the final multiple regression model, we have found that dyslipidemia is a significant determinant of the Impacts score, indicating that disruption of lipid metabolism may worsen the severity of distress disorder in asthma. The possible contribution of dyslipidemia to the pathogenesis of asthma has been suggested previously. For example, dyslipidemia is known to be associated with increased asthma prevalence, moreover, based on results from preclinical (Gowdy and Fessler, 2013) and clinical (Fenger et al., 2013) studies, it has been implicated as a risk factor for asthma. Corroborating findings were also reported by Rastogi et al. who found that dyslipidemia is a predictor of pulmonary function deficits (Rastogi et al., 2015).

A limitation of the present study is that severity of distress disease was quantified by the Impacts score of SGRQ given that exact identification of anxiety or mood disorders according to DSM-V would have caused extra inconvenience for the asthma outpatients and might have been difficult due to the great overlap between symptoms that may be secondary to the mental disorders or asthma *per se* (Porsbjerg and Menzies-Gow, 2017). Nonetheless, the robust correlation between depression and Impacts score has been previously acknowledged (Jones et al., 1992), giving way to the proposition that irisin may serve as a link between altered irisin/BDNF path and evolution of distress disorder.

The lack of post-bronchodilator whole-body plethysmography may also be viewed as a limitation. However, our patient population received asthma control therapy for 15 (IQR: 10–20) years, and patients were instructed to take their medication as usual in the morning of their examinations. Thus, our findings must be interpreted as on treatment results.

Finally, merits of the current work should be articulated like the relatively large clinical sample, implementation of specific instruments (whole-body plethysmography and SGRQ, a validated disease-specific questionnaire), and stringent data analysis.

## Conclusions

The main finding of the present study is that alteration of irisin/BDNF axis parallels the evolution of distress disorder in asthma. To the best of our knowledge, the current study is the first to assess the relationship among serum irisin level, serum BDNF level and the occurrence (and severity) of distress disorder in asthma. The strong positive association between the reciprocal of serum irisin and Impacts score (i.e., the inverse proportionality between serum irisin and Impacts score) in the multiple linear regression model indicates that circulating irisin is associated with a lower occurrence and/or milder extent of distress disorder. Our further finding that irisin's effect is more pronounced if serum BDNF level is higher adds clinical evidence to prior observations in preclinical models regarding this pathway. Nevertheless precise mechanisms should be elucidated in the future. Summarizing, irisin may contribute to BDNF's ability to influence mood and anxiety by altering contextual learning in structures under BDNF control thus induce the evolution of distress disorder.

## Availability of data and material

Database underlying results of the present study is being on further evaluation, thus it is not freely available yet.

## Author contributions

KP, AM, AF, CP, and MES: participated in the patient recruitment and collection of clinical data; LK: helped to perform the statistical analysis; VV and IS: carried out the irisin and BDNF measurement; GT and RG: participated in the design of the study and helped to draft the manuscript; MS: coordinated the patient recruitment and clinical data collection; JZ: conceived of the study, performed the statistical analysis and completed the manuscript; All authors read and approved the final manuscript.

### Conflict of interest statement

The authors declare that the research was conducted in the absence of any commercial or financial relationships that could be construed as a potential conflict of interest.

## Abbreviations

ACOS: asthma-COPD overlap syndrome
apoA1: apolipoprotein A1
apoB: apolipoprotein B
ATS: American Thoracic Society
BDNF: brain derived neurotrophic factor
BTPS: body temperature and pressure saturated
CI: 95% confidence interval
CK: creatine kinase
COPD: chronic obstructive pulmonary disease
CRP: C-reactive protein
ELISA: enzyme-linked immunosorbent assay
ERS: European Respiratory Society
FEF25-75% % pred: forced expiratory flow between 25% and 75% of FVC, percent of predicted value
FEV1% pred: forced expiratory volume in 1 second, percent of predicted value
FEV1/FVC% pred: ratio of FEV1 to FVC (= FEV1% = Tiffeneau index), percent of predicted value
FNDC5: fibronectin type III domain-containing protein 5
FVC% pred: forced vital capacity, percent of predicted value
GINA: Global Initiative for Asthma
GOT: glutamate-oxaloacetate transaminase
GPT: glutamate-pyruvate transaminase
HDL: high-density lipoprotein
HgA1c: hemoglobin A1c (glycated hemoglobin)
HOMA: homeostasis model assessment
IQR: interquartile range
LDH: lactate dehydrogenase
LDL: low-density lipoprotein
Lp(a): lipoprotein (a)
R_aw_: airway resistance
RV% pred: residual volume, percent of predicted value
RV/TLC% pred: ratio of RV to TLC, percent of predicted value
SD: standard deviation
SGRQ: St. George's Respiratory Questionnaire
STROBE: strengthening the reporting of observational studies in epidemiology
sTSH: thyroid-stimulating hormone determined in a sensitive manner
TLC% pred: total lung capacity, percent of predicted value
γGT: gamma-glutamyl transferase.
